# Subcutaneous implantation after endoscopic and traditional thyroid surgery: a retrospective case report

**DOI:** 10.3389/fonc.2024.1412466

**Published:** 2024-06-07

**Authors:** Tingting Zhang, Zhaoxian Ni, Ben Ma, Qinghai Ji, Ning Qu, Rongliang Shi, Yu Wang

**Affiliations:** ^1^ Department of Head and Neck Surgery, Fudan University Shanghai Cancer Center, Shanghai, China; ^2^ Department of Oncology, Shanghai Medical College, Fudan University, Shanghai, China

**Keywords:** thyroid tumor, surgery, endoscopy surgery, implantation, follicular thyroid carcinoma

## Abstract

Subcutaneous implantation is an unexpected complication of thyroid surgery. Our study aimed to analyze the clinical features and outcomes of implantation after thyroid surgery. We retrospectively searched for the patients with implants of thyroid tumor after surgery from our database prior to August 2023. The clinical and pathological data were reviewed. Six female patients with a mean age of 33.6 ± 13.3 years were enrolled in this study. There was a rare case with mucinous adenocarcinoma, three follicular thyroid carcinoma, and two papillary thyroid carcinoma. The case with primary enteric adenocarcinoma of thyroid with subcutaneous implantation was first reported. The patient with mucinous adenocarcinoma received six courses of TP regimen chemotherapy. Five cases received radioactive iodine therapy. After a mean of 69.5 months of follow-up, one case recurred in the lateral region, and no metastasis or recurrence happened in the other five cases. Although the implantation after thyroid surgery is uncommon, the cases serve as a reminder to take greater care to avoid implantation.

## 1 Introduction

In recent years, endoscopic thyroid surgery has been increasingly accepted for its excellent cosmetic advantages. However, improper use of endoscopes can also lead to complications such as necrosis of the surgical approach flap, emphysema, and hypercapnia. Subcutaneous implantation of thyroid tissue or tumor during thyroid surgery has been reported occasionally. There was no epidemiological information on postoperative implantation (1). Although implantation of thyroid tissue or tumors after endoscopic surgery is uncommon, it can spoil a patient’s aesthetic hopes and increase the burden of reoperation.

In this paper, we reported a series of cases who underwent reoperation in our center with implantation after endoscopic and traditional thyroid surgery. We analyzed the characteristics of subcutaneous implantation after thyroid surgery and put forward the treatment and preventive measures.

## 2 Case description

We retrospectively searched for the patients with thyroid tissue or tumor implantation after endoscopic/conventional thyroid surgery who underwent reoperation at the Head and Neck Surgery Department of Fudan University Shanghai Cancer Center before August 2023. The included patients met the following criteria: 1) primary thyroid cancer treated with surgery, 2) with suspicious implantation foci detected by ultrasonography (US) or cytologically confirmed, and 3) amenable to re-surgery and free of serious disease with informed consent for surgery. All tissue specimens were diagnosed by an experienced pathologist.

Six patients with implants of thyroid tumor were collected from our database at FUSCC (**Table 1**). We reviewed the medical records for the clinical and pathological data and followed up the patients through outpatient services and telephone consultations. All the procedures performed in our study were in accordance with the ethical standards of our institutional research committee and the 1964 Helsinki declaration and its later amendments or comparable ethical standards.

**Table 1:** Implantation cases after endoscopic and traditional thyroid surgery in our center.

Case	Gender(y)	Age^a^	Operation	Interval ^b^(month)	Pathology of thyroid lesions and implants	Number ^c^	Location ^d^	Treatment ^e^	Follow-up (m)	Prognosis
1	Female	40	Transaxillary endoscopic thyroidectomy	3	enteric adenocarcinoma	Multiple	Subcutaneous right neck and axilla	Surgery+ chemotherapy	30	NED
2	Female -	57	Transaxillary endoscopic right hemithyroidectomy	30	FTC (malignant)	Multiple	Subcutaneous Right neck/thyroid bed	Surgery+I^131^	72	Bilateral lateral recurrence
3	Female -	31	Endoscopic right hemithyroidectomy via breast	60	FTC (malignant)	Single	subcutaneous right chest	Surgery+I^131^	35	NED
4	Female-	19	Endoscopic left hemithyroidectomy via breast	8	Follicular variant of PTC	Multiple	Bilateral subcutaneous supraclavicular region	Surgery+I^131^	204	NED
5	Female -	26	Right hemithyroidectomy+CLND	48	PTC	Multiple	subcutaneous anterior cervical region	Surgery+I^131^	48	NED
6	Female	29	Transoral endoscopic thyrohyoid cyst excision	6	PTC	Multiple	submental and submandibular region	Surgery+I^131^	28	NED

a Age at first operation.

b Interval between surgery and implantation.

c Number of implanted lesions.

d Location of the implanted lesion.

e Treatment after implantation.

FTC, follicular thyroid carcinoma; PTC, papillary thyroid carcinoma; NED, no evidence of disease.

### 2.1 Case 1

**Figure 1:**
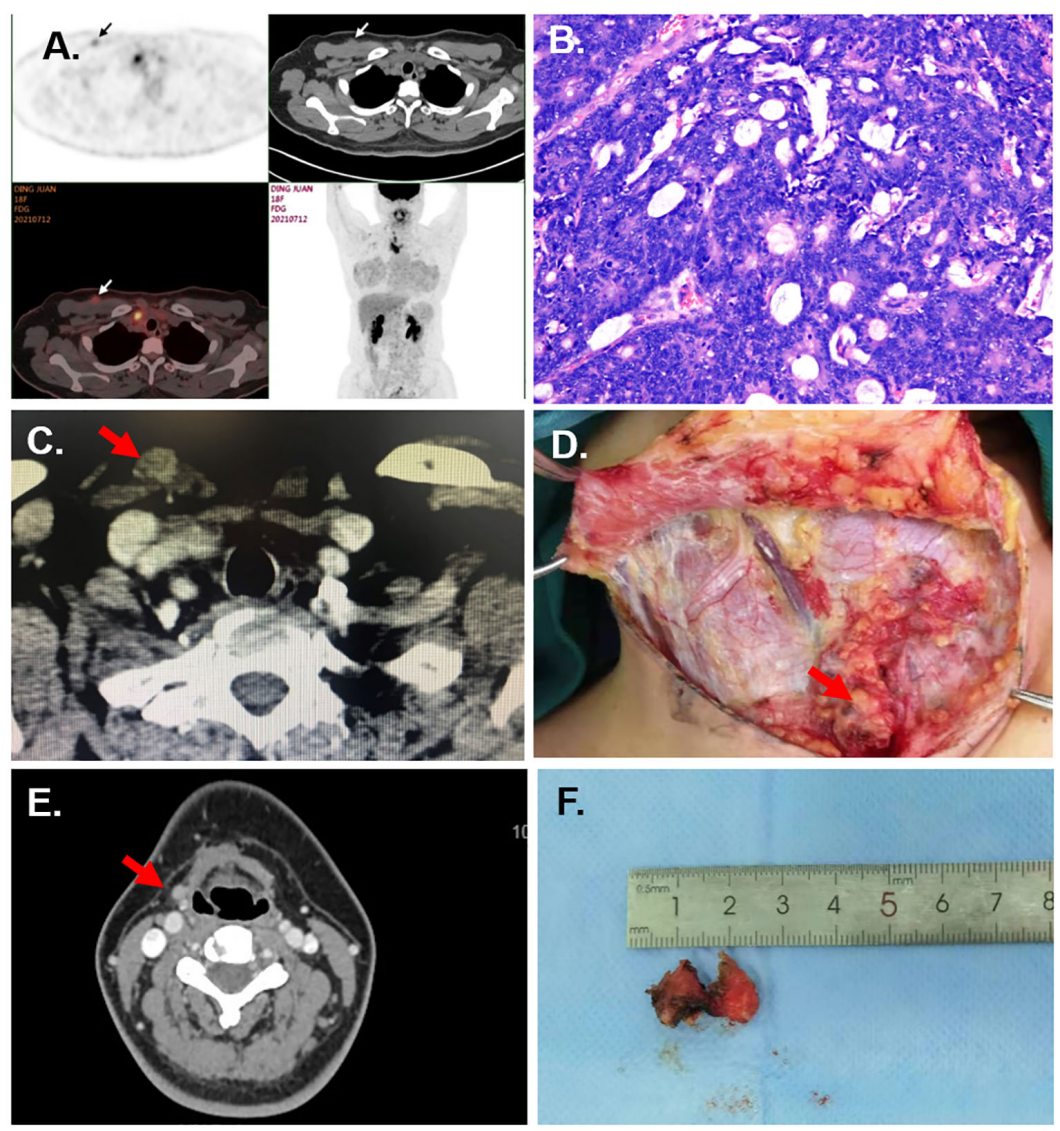
Case information. **(A)** The PET/CT of Case 1 showed the subcutaneous nodules at the right supraclavicular region and axilla. **(B)** Pathological section of implanted lesion of Case 1 (HE staining, under ×20 microscope). **(C)** The CT of Case 2 shows a subcutaneous nodule at the superior of the sternal head of the sternocleidomastoid. **(D)** The multiple subcutaneous implanted lesions are shown in the surgery of Case 5. **(E)** The CT of Case 6 shows the implanted nodules at the right submandibular region. **(F)** The excised tumor implanted at the submental region for Case 6.

This was a rare case with pathological type of thyroid cancer, which had never been reported before. A 40-year-old woman underwent transaxillary endoscopic thyroidectomy for a right thyroid nodule at a local hospital. The pathological section showed an enteric adenocarcinoma. Three months later, she presented to our hospital with ultrasound findings of lesions in the neck and axilla. Pathology consultation at our center confirmed that the initial surgical specimen was enteric adenocarcinoma. Immunohistochemical analysis showed AE1/AE3(+), CK20(+), CK7(+), TTF-1(−), Syn(−), Calcitonin(−), SATB2(+), and CDX2–88(+). PET/CT showed implantation of lesions located in the right neck, axilla, and mediastinum, and no suspicious lesions were detected in any other part of the body (**Figure 1A**). Electronic gastroenteroscopy ruled out the possibility of colorectal or gastric cancer metastasis. Subsequently, the patient underwent a right lateral lymph nodes dissection, and the subcutaneous lesions in the neck and axilla were excised en bloc with the surrounding tissues. The final pathology specimen showed that the enteric adenocarcinoma infiltrated the subcutaneous connective and muscle tissues and metastasized to one lateral lymph node (**Figure 1B**). Then, she received six courses of chemotherapy with TP regimen (docetaxel 120 mg d1+ cisplatin 40 mg d1–3). No metastasis or recurrence has occurred to date.

### 2.2 Case 2

A 57-year-old woman complained of a palpable subcutaneous nodule found in the suprasternal fossa. Three years ago, she underwent transaxillary endoscopic right hemithyroidectomy with pathological diagnosis of follicular thyroid carcinoma (FTC) at a local hospital. Ultrasound and CT showed a 12 mm × 8 mm subcutaneous nodule at the superior of the sternal head of the sternocleidomastoid (**Figure 1C**). Fine needle aspiration (FNA) of the nodule revealed a follicular neoplasm. Subsequently, she underwent bilateral residual lobectomy, central lymph node dissection, and resection of the implanted nodule along with the surrounding tissues. Pathological examination revealed three 0.1–1.0-cm-size nodules in the soft tissue between the sternal head and clavicular head of the sternocleidomastoid muscle, all of which were implanted and metastatic FTCs. Multiple foci of implantation and metastasis were also found in the thyroid bed. After surgery, radioiodine therapy at a dose of 200 mCi was also performed. However, due to recurrence of the bilateral lateral cervical lymph nodes, she underwent a third operation 2 years later. Currently, the patient’s prognosis is favorable.

### 2.3 Case 3

A 31-year-old woman complained of a palpable subcutaneous nodule found on the right chest. Five years ago, she underwent endoscopic right hemithyroidectomy via chest–breast approach at another hospital with pathological diagnosis of “follicular thyroid adenoma”. Pathology consultation at our center showed that the initial surgical specimen was a micro-invasive variant of follicular thyroid carcinoma. FNA revealed a neoplastic lesion. Thus, we removed the lesion from the right chest en bloc along with the surrounding tissues and performed a left lobectomy at our center. After surgery, radioactive iodine therapy at a dose of 100 mCi was administered. No metastasis or recurrence occurred in the 7-year follow-up.

### 2.4 Case 4

A 19-year-old female patient underwent endoscopic left hemithyroidectomy via chest–breast approach in another institution and diagnosed with “multinodular goiter” pathologically 8 months earlier. One month before, a nodule measuring 1 cm at the right supraclavicular region was palpated by herself. Then, she came to our hospital for consultation. Pathology consultation showed that the primary tumor was follicular variant of papillary thyroid carcinoma. Ultrasound showed a 15 mm × 9 mm subcutaneous nodule at the left supraclavicular region and a 12 mm × 8 mm subcutaneous nodule at the right supraclavicular region. FNA revealed palpable nodules as metastatic adenocarcinoma. Subsequently, the patient underwent right thyroidectomy and central lymph nodes dissection, and the nodules in the bilateral subcutaneous supraclavicular region were excised en bloc with the surrounding tissue. The final pathological specimen reported that the bilateral lesions were a follicular variant of papillary thyroid carcinoma. Then, she received a radioactive iodine therapy at a dose of 100 mCi. After a 15-year follow-up, no metastasis or recurrence happened.

### 2.5 Case 5

A 26-year-old female patient had a right hemithyroidectomy and central lymph nodes dissection for PTC pathologically 4 years ago. Six months before, multiple nodules at the neck were palpated by herself. Ultrasound and CT revealed multiple parenchymal nodules in the right paratracheal region, subcutaneous supraclavicular region, anterior trachea, and right lateral region. FNA revealed the nodule as malignant tumor. Then, the patient underwent left hemithyroidectomy, central lymph nodes dissection, and right lateral lymph nodes dissection, and the subcutaneous lesions were excised en bloc. During the surgery, multiple subcutaneous implanted lesions were found (**Figure 1D**). The final pathology specimen showed that PTC infiltrated connective and muscle tissues and metastasized to the lateral lymph nodes. Postoperative radioactive iodine therapy at a dose of 200 mCi was performed. No metastasis or recurrence happened occurred in the 4-year follow-up.

### 2.6 Case 6

Here, a 29-year-old woman underwent transoral endoscopic excision of thyrohyoid cyst 6 months before. However, the pathological section showed papillary thyroid carcinoma (PTC). She presented to our hospital with palpable lesions in the subglottic and submandibular regions. Ultrasound and CT revealed multiple subcutaneous nodules in the submental and submandibular region and multiple enlarged lymph nodes in both lateral regions (**Figure 1E**). FNA proved the nodule as PTC. Then, the patient underwent total thyroidectomy, both lateral and central lymph nodes dissection, and partial hyoid resection, and the subcutaneous lesions were excised en bloc with the surrounding tissue. The final pathology specimen revealed that PTC infiltrated in the connective and muscle tissue and metastasized to the lateral lymph nodes (**Figure 1F**). Postoperative radioactive iodine treatment at a dose of 200 mCi was also performed. No metastasis or recurrence happened occurred during the 2-year follow-up.

## 3 Discussion

There is a growing number of patients choosing endoscopic thyroid surgery for its significant advantages in cosmetic outcome. However, scarless endoscopic thyroidectomy (SET) is scarless only in the neck and may cause greater trauma to the subcutaneous tissue (1). Compared with the traditional surgery, endoscopic thyroid surgery always has several limitations, such as the narrower operative space, longer operation time, and larger subcutaneous space. The thyroid gland is relatively small in size, anatomically rich in blood supply, and has fragile tissues that are easily lacerated2. These anatomical features and endoscopic limitations make the chance of tumor exposure and implantation during thyroid surgery higher, especially endoscopic thyroid surgery (2, 3). Hence, endoscopic thyroid surgery must be performed with strict adherence to the indications for the procedure.

The following factors may account for the implantation of thyroid tissue: 1) unskilled and improper operation, which is the most critical factor (4, 5); 2) improper intraoperative manipulation, which could lead to rupture and spillage of tumor; 3) direct implantation caused by local trauma; 4) contamination of instruments; 5) chimney effect; 6) CO**_2_** insufflation could increase the intracavitary pressure, which may cause port-site tumor implantations (6); 7) aerosolization of tumor cells; and 8) histological characteristics of tumor cells.

Implantation after both malignant and benign thyroid surgery has been reported (7, 8). The incidence of thyroid implantation appears to be associated with the histopathological invasiveness. Papillary and follicular carcinomas have different biological behaviors. PTC has the nature of indolent, while FTC has the higher rate of invasiveness and metastasis (9). It seemed that follicular tumors accounted for an unexpected proportion in the implantation of thyroid surgery. As known, PTC accounts for more than 80% of thyroid cancer as the most common histological type, while FTC represents just 5% (10). However, in six cases that we reported, two cases were diagnosed FTC and another case was follicular variant of papillary thyroid carcinoma. Moreover, cervical soft tissue recurrence of differentiated thyroid carcinoma was reported to be a predictor for distant recurrence (PMID: 28919092).

Hayashi et al. put forward three hypotheses on needle tract implantation following FNA of thyroid cancer: 1) histological transformation occurred spontaneously at the site of tumor cells deposit; 2) tumor–host microenvironment interactions may also induce the occurrence of histological transformation; and (3) aggressive histological cell subgroups may have higher survivability to seeding along the tracks of the fine needle inserted (11). These hypotheses could extend to explain port insertion site implantation after endoscopic surgery. It follows then that those aggressive variants of thyroid cancer may have a higher probability of implantation after endoscopic thyroid surgery.

Avoiding implantation during initial surgery is the most important event. Rigorous and comprehensive preoperative evaluation should be performed. The indications for endoscopic surgery should be strictly followed. Intraoperative manipulation should be meticulous to fit the principles of an-neoplasia surgical operation. It should be highlighted to avoid nodule rupture (by using specimen bags), even if it the nodules are benign (2, 3, 7, 8, 12).

What should be done for implantation after thyroidectomy? Three treatment measures are summarized as follows. First is reoperation. It should include surgical excision of all nodules and unilateral partial lobectomy. Surgery is the most effective method, however, with a high recurrence rate (3, 13, 14). Li and Kim reported a case of implanted nodules that recurred around the operation bed several months after the removal of the nodules, respectively (2, 7). Second is radioiodine treatment for differentiated thyroid carcinoma (DTC). If the implanted nodules have strong property of iodine uptake, patients can receive I131 therapy. The nodules will gradually be absorbed after that (3, 13, 14). Third is the endocrine suppression therapy for DTC. The premise of effective TSH suppression treatment with levothyroxine is the implanted nodules dependent on TSH for growth (3, 13, 14). There are two optional but controversial measures. First is ablation. Ultrasound-guided percutaneous microwave ablation of thyroid nodules is currently used for the treatment of benign primary thyroid nodules. In the future, it may also be considered for the treatment of benign implanted nodules after endoscopic thyroid surgery. Second is follow-up. In the case of implantation after endoscopic surgery reported by Kyung Won Koh, the subcutaneous recurrent nodules are stable under close observation without any other concrete management (8).

## 4 Conclusion

Endoscopic thyroid surgery is drawing more and more attention because of its excellent cosmetic result. Although the implantation after endoscopic and traditional surgery is uncommon, it will undermine a patient’s cosmetic hopes and increase the risk of recurrence. Both benign and malignant thyroid surgery could implant. The incidence of thyroid implantation after open surgery and endoscopic surgery appears to be associated with the histopathological invasiveness, especially for the follicular type. Rigorous and comprehensive preoperative evaluation should be performed. The indications for endoscopic surgery should be strictly followed.

## Data availability statement

The raw data supporting the conclusions of this article will be made available by the authors, without undue reservation.

## Ethics statement

The studies involving humans were approved by the Ethical Committee of Fudan University Shanghai Cancer Center. The studies were conducted in accordance with the local legislation and institutional requirements. Written informed consent for participation was not required from the participants or the participants’ legal guardians/next of kin in accordance with the national legislation and institutional requirements. Written informed consent was obtained from the individual(s) for the publication of any potentially identifiable images or data included in this article.

## Author contributions

TZ: Writing – original draft, Formal analysis. ZN: Writing – original draft, Data curation. BM: Writing – original draft, Formal analysis, Data curation. QJ: Writing – review & editing, Resources, Formal analysis. NQ: Writing – original draft, Resources, Formal analysis. RS: Writing – review & editing, Formal analysis, Conceptualization. YW: Writing – review & editing, Resources, Funding acquisition, Formal analysis, Conceptualization.
